# Legal and ethical framework for global health information and biospecimen exchange - an international perspective

**DOI:** 10.1186/s12910-020-0448-9

**Published:** 2020-01-21

**Authors:** Lara Bernasconi, Selçuk Şen, Luca Angerame, Apolo P. Balyegisawa, Damien Hong Yew Hui, Maximilian Hotter, Chung Y. Hsu, Tatsuya Ito, Francisca Jörger, Wolfgang Krassnitzer, Adam T. Phillips, Rui Li, Louise Stockley, Fabian Tay, Charlotte von Heijne Widlund, Ming Wan, Creany Wong, Henry Yau, Thomas F. Hiemstra, Yagiz Uresin, Gabriela Senti

**Affiliations:** 1 Clinical Trials Center, University Hospital Zurich, Zürich, Switzerland; 2 Center of Excellence for Clinical Research, University of Istanbul, Istanbul, Turkey; 3 Clinical Trial Center Spa, Fondazione Policlinico Universitario A. Gemelli IRCCS, Università Cattolica Sacro Cuore, Rome, Italy; 4 Infectious Diseases Institute of Kampala, Kampala, Uganda; 5 Singapore Clinical Research Institute, Singapore, Singapore; 6 Medical University of Graz, Graz, Austria; 7 Clinical Trial Center, China Medical University Hospital, Taichung, Taiwan; 8 Institute for Advancement of Clinical and Translational Science, Kyoto University and Kyoto University Hospital, Kyoto, Japan; 9 Baim Institute for Clinical Research, Boston, USA; 10 Shanghai Clinical Research Center, Shanghai, China; 11 Cambridge Clinical Trials Unit, Cambridge University Hospitals NHS Foundation Trust, Cambridgem, UK; 12 Karolinska Trial Alliance, Stockholm, Sweden; 13 Clinical Trials Centre, The University of Hong Kong, Pok Fu Lam, Hong Kong

**Keywords:** Data sharing, Biobanking, Big data, Research policies, International exchange

## Abstract

**Background:**

The progress of electronic health technologies and biobanks holds enormous promise for efficient research. Evidence shows that studies based on sharing and secondary use of data/samples have the potential to significantly advance medical knowledge. However, sharing of such resources for international collaboration is hampered by the lack of clarity about ethical and legal requirements for transfer of data and samples across international borders.

**Main text:**

Here, the International Clinical Trial Center Network (ICN) reports the legal and ethical requirements governing data and sample exchange (DSE) across four continents. The most recurring requirement is ethical approval, whereas only in specific conditions approval of national health authorities is required. Informed consent is not required in all sharing situations. However, waiver of informed consent is only allowed in certain countries/regions and under certain circumstances. The current legal and ethical landscape appears to be very complex and under constant evolution. Regulations differ between countries/regions and are often incomplete, leading to uncertainty.

**Conclusion:**

With this work, ICN illuminates the unmet need for a single international collaborative framework to facilitate DSE. Harmonising requirements for global DSE will reduce inefficiency and waste in research. There are many challenges to realising this ambitious vision, including inconsistent terminology and definitions, and heterogeneous and dynamic legal constraints. Here, we identify areas of agreement and significant difference as a necessary first step towards facilitating international collaboration. We propose the establishment of a working group to continue the comparison across jurisdictions, create a standardised glossary and define a set of basic principles and fundamental requirements for DSE.

## Background

Over recent years, topics like “big data”, “biobanks” and “data sharing” have gained momentum in the health science community. The widespread adoption of electronic health records has raised the interest of researchers in exchanging clinical data for secondary use [1]. Patient data can be merged on a large scale for epidemiological studies or disease monitoring [2, 3]. Besides health information technology, biobanks of human specimens have a growing role in the creation of actionable knowledge in health science [4, 5]. Over the last three decades, these repositories have evolved, and the information associated with biological samples has increased enormously [6]. Multiple international initiatives are currently promoting collaboration between existing biobanks to enable larger research projects [7–10].

Despite the increasing opportunities for the advancement of biomedical research through DSE and the availability of data/sample to exchange, this promise has not yet been fully realised through an actual increase in data and sample sharing [11]. This mismatch may be explained by the obstacles presented to researchers of having to navigate the regulatory framework governing the sharing of data/samples. International exchange can be particularly challenging due to conflicting regulations and overlapping terminologies [2, 12]. Adding further complexity, regulations continue to evolve. Some tools have been developed to support researchers, but many cover a limited geographical region or are not sufficiently mature or current [2, 13–15].

Legal restrictions are not the only barriers to seamless DSE. Among others, further limiting factors may be the reluctance of investigators who are aiming to publish first, as well as the interests of research funders. However, we believe that a clearer regulatory landscape would help to overcome also this hindrance to fully enable robust, ideally patient-controlled DSE [16]. In this article, the International Clinical Trial Center Network (ICN) [17], a network that brings together top-tier experience and knowledge of 19 clinical trial centres from across the globe, draws a simplified picture of the regulatory requirements to exchange health data and biospecimens across four continents and highlights differences that could influence the conduct of international projects. The ICN brings together different non-profit institutions with the common objective to encourage continuous international dialogue, to provide guidance to researchers, and to promote international collaboration. Relevant terms concerning data protection are also compared. This work should help to advance the global exchange of data and samples as well as to support the development of a valuable tool that helps researchers in large-scale international research projects.

## Main text

We surveyed members of the ICN between July 2018 and October 2019, collecting detailed information on local guidelines, regulations and restrictions concerning the international exchange of data and samples, centred on 3 out of the 4 key dimensions of health information exchange defined by Holmgren and colleagues [18].

1. *What defines the “Rules” of Exchange?*

We collected information on policies applying in the context of international DSE and authorities involved in its approval.

2. *Who is exchanging? Relationship between exchanging partners*

Special requirements/restrictions concerning data/samples receivers (e.g. specific countries or for-profit organizations) were assessed.

3. *What is exchanged? Types of information*

Here, we focused specifically on the further use for research purposes of already available clinical data/samples rather than the collection of new data/samples, distinguishing between: a) genetic data, non-genetic data and biological samples; b) different data formats; and c) different levels of subject’s privacy protection. The present report is not concerned with data format.

Thirteen centres from eleven countries contributed to data gathering (ISO 3166 code in brackets): Austria (AUT), China (CHN), United Kingdom (GBR), Italy (ITA), Japan (JPN), Singapore (SGP), Sweden (SWE), Switzerland (CHE), Turkey (TUR), Uganda (UGA) and United States of America (USA). Hong Kong (HKG) and Taiwan (TWN) are described as separate regions since they have local regulations governing the international transfer of data/samples that differ from Mainland China. The regulatory requirements collected for the United Kingdom are applicable for England, Northern Ireland, Scotland and Wales. However, different health authorities may be involved in the approval of specific research projects. For each participating ICN country/region, a matrix showing the applicable requirements in specific sharing situations was created. Differences between the regulatory frameworks of represented countries/regions were identified and are reported in this article, together with differences in the terms related to data protection.

### The regulatory framework is in continuous evolution

For all participating countries/regions, this article provides a cross-sectional appraisal of the current situation, recognising continuous evolution that requires frequent revision. In some countries/regions, a number of regulatory aspects are not currently precisely defined. At the time of writing, a certain degree of regulatory uncertainty also affects the European Union (EU). On 25th May 2018, the General Data Protection Regulation (GDPR) was enforced within EU. The GDPR creates new exemptions for research and leaves some room to member states to specify their own rules [19]. In response, many EU member states are reviewing national regulations governing data protection in the field of clinical research, addressing areas in which flexibility is permitted by the GDPR.

According to the GDPR, personal data may only be transferred to third countries if an adequate level of data protection within the meaning of Art. Forty-five is guaranteed in the receiving country or if the data transfer is subject to an exception pursuant to Art. 49. Thirteen adequacy decisions have been adopted so far, as reported on the website of the European Commission. Of the countries/regions participating in this project, only Japan, Switzerland, and the USA (limited to the Privacy Shield framework) have been recognized as providing adequate protection.

### Data protection terminology is not consistent across countries/regions

In all participating countries/regions, information which may be used to identify a specific person indirectly is also considered personal data. However, in Japan and Singapore, coded information can be regarded as non-personal information where the access to the original personal information is unlikely or prevented. Interestingly, the Japanese definition specifically distinguishes between different personal information recording forms (documents, drawings or electromagnetic records). The latter includes electronic, magnetic and other recording forms not otherwise specified that cannot be recognized directly through the human senses. In Singapore, all data pertaining to an individual, whether true or false, is considered personal data. In Hong Kong, Japan and the United Kingdom, deceased persons do not have personal data. This also applies to China and Taiwan, although not directly specified in the local definition of personal data. According to recital 27 of the GDPR, deceased persons do have personal data. However, the regulation only protects living persons. This leaves member states the possibility to regulate the processing of personal data of deceased persons. Austria and Sweden do not provide for special provisions in this regard. In Italy, the rights described in articles 15 to 22 of the GDPR for deceased persons can be exercised by those who have an interest, or act to protect the data subject or for family reasons deserving protection [20]. In Switzerland, deceased persons’ data fall under the doctor-patient confidentiality agreement, but is not considered personal data. Special regulations apply for research with deceased persons, i.e. surrogate consent can be provided by the ethics committee [21]. In the other countries, the respective data protection regulations apply in respect of deceased individuals, but this is limited to 10 years in Singapore and 50 years in the USA [22, 23].

Only the European countries, Singapore and Taiwan could provide a legal definition for the term “pseudonymization” or “coding”. In these countries/regions, pseudonymized (coded) data can no longer be attributed to a specific subject without the use of additional information. Only Singapore distinguishes between reversible and irreversible coding. In the latter case, original values are properly disposed of and the pseudonymisation is done in a non-repeatable fashion. In Mainland China, the term “de-identification” is used when pseudonyms, encryption, hash functions or other technical methods are used to replace identifiers [24]. In the USA, data are considered de-identified if they have been stripped of common identifiers and there is no reasonable basis to believe that the information can be used to identify an individual [25]. Hong Kong, Taiwan and Uganda do not have a single, pervasive legal definition for data which cannot be traced to a specific person, whereas the European countries, China, Singapore and Turkey prefer the term “anonymized”. However, some definitions differ in how strictly the possibility of re-identification is limited. While GDPR and Swiss law set a relatively low bar for “identifiable”, anonymized data should be impossible to link with an individual according to the Turkish law. In all cases, anonymized data are not considered as personal data, nor are de-identified data in the USA.

The Japanese Data Protection Law distinguishes three categories of information: personal information, anonymously processed information and non-personal information [26]. Under other laws such as the GDPR, anonymously processed information and non-personal information correspond to pseudonymized information and anonymized information, respectively.

The only countries/regions having a legal definition for the term “encrypted” are Singapore, Taiwan and the United Kingdom. In the United Kingdom and Singapore, encrypted data are considered as personal data. In Taiwan, encryption is the process of making personal data irreversibly unidentifiable. In other countries/regions, this would probably be considered as anonymization. Of note, Taiwan does not have a definition for the term “anonymized”.

A list of definitions is provided in the supplementary information (see Additional file 1).

### Data privacy level affects transferability

As shown in Table 1, following the respective country-specific regulations it is possible to transfer data/samples with any privacy protection level from Austria, Switzerland, Mainland China, United Kingdom, Hong Kong, Japan, Sweden, Taiwan and USA [19, 21, 27–36]. In the other countries/regions, including Italy as the only European representative, the transfer of data/samples containing directly identifiable information (i.e. uncoded data/samples) is not permitted [20, 22, 37–40]. According to the Turkish Data Protection Law, uncoded data/samples can be transferred with participants’ consent [41]. However, regulations on clinical research and personal health data state that personal data must be coded or anonymized for privacy [38, 42, 43].

**Table 1:** Transferability of data/samples with different level of subject’s privacy protection

	AUT	CHE	CHN	GBR	HKG	JPN	ITA	SGP	SWE	TUR	TWN	UGA	USA
Transferability													
Uncoded data/sample	yes	yes	yes^a^	yes	yes	yes^b^	no	no	yes	no	yes	no	yes
Coded data/sample	yes	yes	yes^a,b^	yes	yes	yes^b^	yes	yes	yes	yes	yes	yes	n.a
Anonymized data/sample	yes	yes	yes^a^	yes	yes	yes^b^	yes	yes	yes	yes	yes	yes	yes^b^

*n.a* “not applicable”, this type of data/samples is not defined in the law

^a^The cross-border transfer of non-genetic data is not regulated but the transfer is possible

^b^Different terms from coded/anonymized are used to categorize subject’s privacy protection (see above)

### Informed consent may not be always required

As shown in Table 2, informed consent is always required to transfer any data/samples for research from Mainland China, Italy and Singapore. This also applies to Japan, although the requirements for biological samples are not fully defined yet. In Turkey, Hong Kong, Taiwan, USA and the European countries except Italy, informed consent is not required to transfer anonymized data. In these same countries, except for Austria, informed consent is not required for the transfer of anonymized biological samples either. However, in Switzerland and Turkey, the patient has to be informed about the planned anonymization of biological samples and has the right to refuse (opt-out consent) [21].

**Table 2:** Requirements for informed consent (IC) to transfer data/samples abroad

	AUT	CHE	CHN	GBR	HKG	JPN	ITA	SGP	SWE	TUR	TWN	UGA	USA
IC required to transfer													
Uncoded data/samples	yes	yes	yes	yes^b^	yes	yes^d,e^	n.a.	n.a.	yes^b^	n.a.	yes	n.a.	yes
Coded data/samples	yes	yes^b^	yes^e^	yes^b^	yes	yes^d,e^	yes	yes	yes^b^	yes	yes	yes^b^	n.a.
Anonymized data/samples	yes^a^	no^c^	yes	no	no	yes^d,e^	yes	yes	no	no^c^	no	yes^b^	no^e^

*n.a* “not applicable”, this type of data/samples is not transferable or is not defined in the law

^a^Biological samples only

^b^Waiver of IC allowed under certain conditions

^c^Patient has to be informed about the planned anonymization of biological samples and has the right to refuse

^d^Not regulated for biological samples

^e^Different terms from coded/anonymized are used to categorize subject’s privacy protection (see above)

In Uganda, Switzerland and the United Kingdom, waiver of informed consent is permitted by Ethic Committee and applicable regulatory authority in situations where it is problematic or challenging to obtain consent or where the public interest of research outweigh the interests of the subjects [21, 37, 44, 45]. In the United Kingdom, an additional approval from a health authority is required in these cases [44]. In Sweden, research may be carried out without informed consent if weakened state of health prevents the subject from expressing an opinion. There is to be consultation with the patient’s closest relatives and a custodian or other legal representative. However, proxy consent is not required, as only the research subject (if > 18 years old) can give consent in this country [46].

### National health authorities are involved less often than ethics committees

A comparison of the required approvals is displayed in Table 3. In countries/regions where informed consent is not required to transfer anonymized data/samples, approval from the ethics committee is not required either, except for Turkey. In this country, ethical approval but no informed consent is required to transfer anonymized data/samples. In the United Kingdom, ethical approval is only required to transfer coded and uncoded data/samples for specific research projects. This also applies to Switzerland where, furthermore, approval is only required if the Swiss institution transferring the data/samples is involved in the project. The creation of databases and biobanks does not require approval in neither of the two countries just mentioned [21, 47]. However, British researchers have the option to seek a five-years lasting generic ethical approval for a range of research within the conditions of the ethical approval [47]. In all other countries, ethical approval is always required to transfer coded and uncoded data/samples.

**Table 3:** Required approvals to transfer data/samples abroad

	AUT	CHE	CHN	GBR	HKG	JPN	ITA	SGP	SWE	TUR	TWN	UGA	USA
Ethical approval required													
Uncoded data/samples	yes	yes^a,b^	yes	yes^a^	yes	yes^f^	n.a.	n.a.	yes	n.a.	yes	n.a.	yes
Coded data/samples	yes	yes^a,b^	yes^f^	yes^a^	yes	yes^f^	yes	yes	yes	yes	yes	yes	n.a
Anonymized data/samples	yes^e^	no	yes	no	no	yes^f^	yes	yes	no	yes	no	yes	no
Approval from national health authority required													
Uncoded data/samples	no	no^c^	yes^d^	yes^a^	no	no^f^	no	n.a.	yes^e^	n.a.	yes	n.a.	no
Coded data/samples	no	no^c^	yes^d,f^	yes^a^	no	no^f^	no	no	yes^e^	yes	yes	yes	n.a
Anonymized data/samples	no	no^c^	yes^d^	yes^a^	no	no^f^	no	no	no	yes	yes	yes	no

*n.a* “not applicable”, this type of data/samples is not transferrable or is not defined in the law

^a^When transferred for specific research projects. Not for creating databases or biobanks

^b^Approval is only required if the institution transferring the data/samples is involved in the project

^c^Unless data/samples are transferred to a country without adequate protection

^d^Biological samples and genetic data only

^e^Biological samples only

^f^Different terms from coded/anonymized are used to categorize subject’s privacy protection (see above)

In Austria, Hong Kong, Italy, Japan, Singapore and the USA, no national health authority is involved in the international transfer of data/samples for research purposes. In contrast, Uganda, Taiwan and Turkey require the approval of the local health authority (Uganda National Council for Science and Technology, the Ministry of Health and Welfare and Ministry of Health, respectively) in all sharing situations [37, 43, 48]. In the remaining countries/regions, health authorities are only involved in certain types of transfer.

## Conclusions

The heterogeneous nature and complexity of several diseases and the deep characterization of individuals have led to the rapid development of personalized medicine. Access to large amount of health data is the next challenge for this medical model [49]. Where the available data in one country may not be sufficient to efficiently develop new pharmacogenetic-based treatment strategies and new algorithms to diagnose patients, DSE represents a huge resource to gain validity of findings and increase the impact of research. However, the lack of a unified ethical and legal environment presents an obstacle for the scientific community [2, 12]. The present work demonstrates that researchers currently operate within a very complex legal and ethical landscape. Regulations differ between countries/regions and are often incomplete, leading to uncertainty. Differences can also be found between regulations within a single country. In Turkey for example, although the data protection law allows the usage of uncoded data with subjects’ consent, sub-regulations restrict the usage of uncoded data relating to health. In addition, the rapid evolution of regulations over time make it impractical for researchers to keep abreast of the latest regulations across all jurisdictions. There is an unmet need for an accessible resource that provides information on country-specific requirements for DSE. Despite this, the complexity of this field means that in many cases, researchers may still require the guidance of experienced professionals [12]. This might be facilitated by International networks of research institutions such as ICN.

The most universal requirement for transferring data or samples across national borders identified in this work is ethical approval, whereas approval of national health authorities is required only in specific settings. Informed consent is not required in all sharing situations. However, waiver of informed consent is only allowed in certain countries/regions and under certain conditions.

When considering individuals’ privacy, the transfer of solely anonymized data may seem to be a practical solution. However, anonymization terminates the ability to link records with new datasets or to re-contact participants. Further, some argue that complete anonymization is impossible as long as the original records still exist [2, 50], leading to concerns over potential data misuse and compromised privacy. This work shows that transfer of anonymized data may also not be subject to less stringent regulation.

The ideal would be a single unified global legal and ethical framework to which all countries/regions could subscribe and that would permit seamless data and sample exchange. Sets of ethical principles for medical research already exist, such as the Declaration of Helsinki, the Declaration of Taipei, the Council for International Organizations of Medical Sciences (CIOMS) international ethical guidelines for biomedical research involving human subjects and the Organization for Economic Co-operation and Development (OECD) Guidelines on human biobanks and genetic research databases. However, these are not legally binding instruments under international law. Discordances can also be found between these internationally recognized guidelines. Given the heterogeneous regulatory and legal landscape reported here, and disagreement even in simple terminology and definitions, much work is needed in order to achieve the above-mentioned goal of a unified framework.

To address the need of global harmonization, we propose a collaborative and interdisciplinary approach trough the establishment of a working group including researchers, patient representatives, information technology experts and legal expertise from different geographical regions. This will require engagement of all relevant stakeholders. The first step will be a further detailed description of the current regulatory landscape, ideally with representation from all regions. This will allow generation of a library of shared and differing terms, identifying areas in greatest need of harmonization. It has become clear through this work that all regions share a desire to adequately protect the rights of the individual in DSE, and that the differences outlined in this manuscript lie in interpretation of data protection and the way in which it is defined. A key step will be agreeing a glossary of standardized terms and definitions. Next, guidance on the principles of responsible data and sample exchange should be agreed and published, allowing governments and regulators to benchmark and harmonize national regulations and laws. Finding the balance between a subject’s right to privacy and the public interest in advancing medical research in a manner that would be widely acceptable will be challenging, as already anticipated by the existing conflicts between current requirements to deposit data in research repositories and the GDPR [51]. The work of other groups such as the Global Initiative for the Ethical Use of Human Specimens (GIFT), who elaborated a set of recommendations for standardizing informed consent, should also be taken into consideration during this phase [52].

We recognise that harmonization of DSE will not in itself solve the limited extent of DSE. Even when DSE is possible, whether sharing takes place depends on many other factors including the attitudes of the investigators, the interests or requirements of research funders and others. However, we believe that a clearer regulatory landscape would help to overcome also this hindrance to fully enable robust, ideally patient-controlled health data/sample usage [16].

Our report is the first to compare the requirements for DSE across multiple jurisdictions spanning four continents and is strengthened by its representativeness. However, there are important limitations, including the lack of representation from South America and Australasia. Furthermore, a comprehensive and in-depth review of all legal considerations for each jurisdiction is beyond the scope of this preliminary survey. Data were provided by relevant ICN members, which represent leading clinical trials centres within the represented jurisdictions. We consulted with legal experts but have not sought input and verification from individual regulators. Additionally, an official English translation of the national regulations does not always exist. There is a need for further detailed work in collaboration with regulators where possible, but we consider the present overview a crucial first step towards identifying obstacles and opportunities from DSE. While we cannot directly mandate change, we hope that this work can form the basis for progress through increasing awareness, standardization and guidelines.

## Supplementary information

**Additional file 1.** List of definitions: the Excel file “List of definitions_data protection.xlsx” includes legal definitions for the terms “personal data”, “anonymized”, “de-identified”, “pseudonymized” and “encrypted”, as provided by the participating ICN countries/regions.

## Data Availability

Not applicable.

## Abbreviations

AUT: Austria
CHE: Switzerland
CHN: China
CIOMS: Council for International Organizations of Medical Sciences
DSE: Data and sample exchange
EU: European Union
GBR: United Kingdom of Great Britain and Northern Ireland
GDPR: General Data Protection Regulation
HKG: Hong Kong
ICN: International Clinical Trial Center Network
ITA: Italy
JPN: Japan
OECD: Organization for Economic Co-operation and Development
SGP: Singapore
SWE: Sweden
TUR: Turkey
TWN: Taiwan
UGA: Uganda
USA: United States of America
